# Transpersonal Caritas Relationship: A new concept from the unitary caring science framework of Jean Watson

**DOI:** 10.17533/udea.iee.v41n3e02

**Published:** 2023-10-02

**Authors:** Mayut Delgado-Galeano, Luz Eugenia Ibáñez-Alfonso, Beatriz Villamizar-Carvajal, María Mercedes Durán de Villalobos

**Affiliations:** 2 Registered Nurse, Master, Titular Professor. Email: libanez@saber.uis.edu.co Universidad Industrial de Santander Colombia libanez@saber.uis.edu.co; 3 Registered Nurse, PhD, Titular Professor. Email: beatriz@uis.edu.co Universidad Industrial de Santander Colombia beatriz@uis.edu.co; 4 Registered Nurse, PhD, Emeritus/Titular Professor. Email: mmvillalobos@gmail.com Universidad Industrial de Santander Colombia mmvillalobos@gmail.com; 5 Health Faculty. Nursing School at Universidad Industrial de Santander, Bucaramanga (Colombia) Universidad Industrial de Santander Universidad Industrial de Santander Bucaramanga Colombia; 6 Nursing Faculty, Universidad Nacional de Colombia. Nursing and Rehabilitation Faculty, Universidad de la Sabana, Bogotá (Colombia) Universidad de la Sabana Nursing and Rehabilitation Faculty Universidad de la Sabana Bogotá Colombia

**Keywords:** nursing theory, nursing care, nursing, teoría de enfermería, atención de enfermería, enfermería, teoria de enfermagem, cuidados de enfermagem, enfermagem

## Abstract

**Objective.:**

To analyze deeply the concept of the transpersonal caring relationship as the core of the theory of Caring Science proposed by Jean Watson. To present a historical evolution and to introduce the Transpersonal Caritas Relationship construct.

**Methods.:**

Methodological Study to support the central concept measured by the Watson Caritas patient instrument. We designed a focus group with four nursing scholars to develop the "Transpersonal Caritas Relationship" construct. We recount the history of the concept of the transpersonal caring relationship, then analyze this concept in terms of Watson's theory. We reviewed the concept with Dr. Jean Watson, presented her with the construct, and discussed our considerations.

**Results.:**

This article introduces a transitional adaptation of the concept of transpersonal relationship to Caritas’ transpersonal relationship. Transpersonal Caritas Relationship is the foundation of evolved Caritas nursing, recognizing that mutual caring affects the universal field we all belong to Caritas' consciousness and action affect the energy field when the nurse relates with the other, making it possible to awaken the compassionate heart, which is the foundation of Evolved Caritas Nursing Universal love and in this way evolve to the Caritas consciousness that allows recognizing the other with loving kindness in the practice of careful. This is the proposed central concept measured in the caring approach using the Watson Caritas Patient.

**Conclusion.:**

This article introduces a transitional adaptation of the concept of transpersonal relationship to the Caritas transpersonal relationship, which is the foundation of Caritas Evolved Nursing**.**

## Resumen

**Objetivo.:**

Analizar de manera profunda el concepto de relación de cuidado transpersonal como núcleo de la teoría de la Ciencia del Cuidado propuesta por Jean Watson. Presentando la evolución histórica e introduciendo el constructo “Relación Transpersonal Caritas”**.**

**Métodos.:**

Estudio Metodológico para sustentar el concepto central medido por el instrumento paciente Watson Caritas. Se diseñó un grupo focal con cuatro académicas de enfermería para desarrollar el constructo "Relación Transpersonal Caritas". Posteriormente se narra la historia del concepto de relación de cuidado transpersonal y luego se analiza este concepto en términos de la teoría de Watson. Se presento y revisó el constructo desarrollado a la Dra. Jean Watson, por último, se discutieron las consideraciones surgidas.

**Resultados.:**

Este artículo presenta una adaptación transitoria del concepto de relación transpersonal a la relación transpersonal de Caritas. La Relación Transpersonal Caritas es la base de la evolución de la enfermería Caritas, reconociendo que el cuidado mutuo afecta el campo universal al que todos pertenecemos. La conciencia y la acción de Caritas afectan el campo energético cuando la enfermera se relaciona con el otro, posibilitando despertar el corazón compasivo, que es la base de la Enfermería Cáritas Evolucionada. Amor universal y de esta manera evolucionar hacia la conciencia caritas que permite reconocer al otro con bondad amorosa en la práctica del cuidado. Este es el concepto central propuesto medido en la práctica del cuidado utilizando el Watson Caritas Patient**.**

**Conclusión.:**

Este artículo introduce una adaptación transicional del concepto de relación transpersonal a la relación transpersonal Caritas, que es el fundamento de la Enfermería Caritas Evolucionada**.**

## Resumo

**Objetivo:**

. Analisar em profundidade o conceito de relação de cuidado transpessoal como cerne da teoria da Ciência do Cuidado proposta por Jean Watson. Apresentando a evolução histórica e introduzindo o construto “Relacionamento Transpessoal da Caritas”.

**Método:**

s. Estudo metodológico para fundamentar o conceito central mensurado pelo instrumento de paciente Watson Caritas. Foi desenhado um grupo focal com quatro acadêmicos de enfermagem para desenvolver o construto “Relacionamento Transpessoal da Caritas”. Posteriormente, é narrada a história do conceito de relação de cuidado transpessoal e este conceito é então analisado à luz da teoria de Watson. O construto desenvolvido foi apresentado e revisado à Dra. Jean Watson e, por fim, foram discutidas as considerações que surgiram.

**Resultados.:**

Este artigo apresenta uma adaptação temporária do conceito de relação transpessoal para a relação transpessoal da Caritas. A Relação Transpessoal da Caritas é a base para a evolução da enfermagem da Caritas, reconhecendo que o cuidado mútuo afeta o campo universal ao qual todos pertencemos. A consciência e a ação da Caritas afetam o campo energético quando o enfermeiro se relaciona com os outros, possibilitando despertar o coração compassivo, que é a base da Caritas Evolved Nursing. Amor universal e desta forma evoluir para a consciência caritas que nos permite reconhecer o outro com bondade amorosa na prática do cuidado. Este é o conceito central proposto medido na prática assistencial por meio do Watson Caritas Patient.

**Conclusão:**

. Este artigo apresenta uma adaptação transitória do conceito de relação transpessoal para a relação transpessoal da Caritas, que é a base da Caritas Evolved Nursing.

## Introduction

The central thesis of Watson's theory of Caring Science is that "human beings cannot be treated as objects and cannot be separated from self, other, nature, and the universe." Therefore, the theory is based on the transpersonal caring that happens on occasion or at the caring moment and the consciousness of caring, which brings us healing.^1^ The philosophical view of the theory of caring human science accords with interconnectivity as the basis of all care interactions. Transpersonal caring considered a fundamental concept of this theory, recognizes the unity of life and care, beginning with the individual and progressing towards the other, the community, the world, planet Earth, the universe and beyond. It follows the Buddhist principle of anatman, or non-self (oneness), affirming, as Chodron, 2014 and Hanh, 1999, that we are all inextricably connected. Through Anatman, we "recognize ourselves in everyone we meet."^2^

The frame of reference of human caring theory is a central part of the nursing discipline. The philosophical, ethical-moral-spiritual stance influences this framework, which is intertwined with the arts, the humanities and fields involving the study and practice of caring for a person to encompass human science's humanitarian focus on processes, phenomena and experiences in human caring.^1^ The above-mentioned falls within a neither dualistic nor rational worldview, where there is connectivity with the Whole, understood as the universal field of the infinite and Cosmic LOVE.^1^ Watson takes up this concept from the Karuna Buddhist tenet, which relates to love and translates as compassion.^3^^-^^6^ This concept has two components: "com," which means union "with," and passion, which means suffering.^2^^,^^3^^-^^6^ According to Hanh, Karuna intends to transform or alleviate the burden of suffering and pain by engaging in elements such as deep concern, empathy and "dwelling in the present moment".^3^^-^^6^ Although suffering is inevitable, we must alleviate it and not let it paralyze us. As Hanh says, we need to allow the present moment's happiness to fill people's hearts, creating peace, joy, and happiness through compassion.^2^

The theory of human science is immersed in the worldview known as the unitary transformative paradigm^7^^-^^8^, non-local consciousness (Dossey 1991) or medicine/nursing into Era III.^1^ This caring model includes a calling for fields such as the arts and sciences, making it a cornerstone that encompasses and interconnects art, science, the humanities, and the nursing profession's spirituality, whose core is the human phenomenon in practice. It is an invitation and an opportunity to internalize and grow by using philosophy as a personal and professional way of life.^9^

On the other hand, loving-kindness and compassion are also frequently mentioned in the healthcare literature. However, these concepts are often rhetorical because they are based on preconceived theoretical definitions and lack specificity, clinical applicability and conceptual validity. They also do not consider how patients experience these concepts or the meaning they give to them, likely because they have received little empirical attention. However, many studies on this matter recently emerged^10^, contributing to a body of evidence supporting this knowledge. As a result, these topics are referred to with the quality-of-care indicators and proposals for humanization in patient care.^10^ Considering what was mentioned above, the conceptual approach of transpersonal care is justified, as is the importance of advancing its understanding and implementing it in different educational and professional performance scenarios.

The transpersonal caring relationship occurs when the provider of care connects with someone and embraces their spirit through genuine and complete care, intending to be present in the here and now and conveying concern for the other person's inner life and the personal meaning that they give to situations, experiences and moments in life.^1^ The degree to which a nurse can detect a person's condition on the level of their soul and spirit, over and above their physical condition, is influenced by and related to the consciousness of universal love and caring intentions, as well as by how the nurse enters in physical space or phenomenal field of the other person.^9^ Being able to go beyond the physical to the consciousness of universal love requires a thorough ontological reflection along the lines of the philosopher Byung Chul Han,^11^ who proposed that "sensitivity and receptivity for the other presupposes an exposure that offers itself even in suffering. It is a pain. Without this primordial pain, the ego becomes more emboldened, exalts his (her) for itself, and objectifies the other by reducing it to an object". Meanwhile, for Levinas,^12^ sensitivity and receptivity for the other presuppose vulnerability. The painful wound is a primordial opening to the other".^12^ In this way, transpersonal caring competencies are related to the promotion of human competencies and to forms of being and becoming a nurse at times when indifference and coldness towards the other are predominant; as Chul Han^11^ says, "our soul is hardened, such that we are not at all sensitive or receptive to the other. Without feeling pain for the other, we have no way to access the other's pain".^11^

In Watson's theory, these competencies and practical knowledge of Caritas caring are essential healing and technological competencies that make it possible to see the human-universe relationship as one. In this way, by developing caring-healing concepts, practices, theories, and philosophies that intertwine with Love, Caring is incorporated into our consciousness and intends to affect the Whole by using our unique gifts and talents in the practice of nursing.^1^ As Watson says, this involves a holographic view of caring, which mirrors the holographic universe. The Whole is in each part, and each piece affects the Whole. In this way, our role -our personal and professional work- contributes to making a difference at the moment and affects the universal holographic field that surrounds us and to which we all belong.^1^ This approach departs from the conventional, modern biomedical model that considers technological curing competencies as fundamental to care and transpersonal caring competencies and concepts as valuable to the practice but not essential.^13^^-^^16^ They also are not part of the healthcare culture or the models current health systems offer.^1^ This article aims to present the Transpersonal Caritas Relationship construct from the perspective of the evolution of the concept proposed by Jean Watson and applied within the theory of Caring Science framework. 

## Methods

The methodological study developed in sequential phases, which aims to support the central concept measured by the Watson Caritas Patient® instrument. 1)First, the history of the concept of Transpersonal Care Relationship is presented; 2) a description of the basis of this concept; 3) a presentation of the construct of the Caritas Transpersonal Relationship; and, finally, 4) our reflections. A focus group was designed with four nursing academic university professors with training and experience in analyzing nursing models and theories to develop the "Transpersonal Caritas Relationship." For this open discussion, one of the teachers led the dialogue, established initial agreements, and clarified the purpose of the meeting. Subsequently, a contrast was presented with situations that helped reflect on everyday practice within a philosophical framework that can be questioned regarding implementation in practice. Next, the history of the "transpersonal care relationship" concept was explained, and it was analyzed in terms of Watson's theory. The document previously developed and analyzed by the authors with the concept "Caritas Relationship" was presented and reviewed. Transpersonal", at this time, we had the participation of Dr. Jean Watson; the meeting focused on discussing the developed concept, capturing the ideas and appreciations in this regard, and, finally, some of our considerations were discussed. The present study did not need ethical approval because no human participants were involved in the present work.

## Results

### Background: The history of the concept of the transpersonal caring relationship

Human caring connection begins with its place in cosmology, ontology, epistemology, ethics, aesthetics and the philosophy of the science of human beings from a unitary perspective. As mentioned before, this refers to what Rogers first proposed as the unitary transformative paradigm^11^^,^^18^, Fawcett^19^ with her proposal of "simultaneous view," followed by Newman ^7^^-^^8^ with the unitary transformative paradigm, and Watson^1^ with "Non-local Consciousness or Era III Medicine/Nursing".^1^ This unitary caring science arose from the transpersonal caring theory and its application through Caritas processes.^17^ Everything is connected at an energetic level in the unitary transformative paradigm, and all acts or actions, no matter how small, affect the energy field. In this paradigm, human beings are seen as a whole with no division, and as Watson explains, caring moments are transpersonal moments that transcend time, space, and physical presence.^1^ The notions shared by this paradigm and human being science mix and bring to life the unitary model of caring science.^17^

For Watson,^1^ the connection between love and caring creates an opening, an alignment with the source of infinite love, the largest source of internal and external healing for oneself and others. Thus, this connection goes deeper than being kind or wishing the best for others. The essential qualities include a feeling of empathy, altruistic action, reverence for equanimity and being in the present moment. Consequently, a moment of transpersonal caring can be considered a transformed turning point for healing.^1^

Watson first introduced the concept of the Transpersonal Caring Relationship in 1979.^20^ Her theory states the role, mission and professional and ethical pact nurses have with society to support human caring and preserve human dignity and integrity amid multiple threats and crises, including the suffering of death.^(20.21)^ It also establishes the interconnection among all people at the human, planetary and universal levels, based on the concept of the Transpersonal Caring Relationship as fundamental to enabling caring and healing from a universal perspective.^22^ In this respect, Watson takes up contributions that renowned academics have made to the definition of Self, such as the work of Carl Rogers, and also uses Mumford's notion of the human center as a basis for developing the concept of the transpersonal process.^17^ Her ideas about transpersonal caring also relate to the meaning of that term in transpersonal psychology, as inspired by Lazarus to expand the field of meaning associated with a one-on-one caring relationship.^22^ Other ideas about transpersonal caring have been promoted by phenomenology, sociology, philosophy and psychology through works by Kierkegaard that were initially circulated in 1846 and published in 1941, by Whitehead in 1953, Chardin in 1967, Giorgi in 1970, Taylor in 1974, Gadow in 1980 and 1984 and Zukav in 1990.^23^

Zen philosophy contributions, with representatives such as the master Thich Nhat Hanh also support Watson. She connects them with caring science by emphasizing two central qualities of compassionate knowledge: an intentional presence and the alleviation of suffering according to the Buddhist view of compassion, not as a religious matter but as a human concern that is essential for our peace and human survival.^2^ According to the Buddha, compassion gives us the means to face and overcome the suffering we experience and find in the world.^2^

Continuing with this historical recounting of the concept of transpersonal relationship, it is crucial to recognize that nursing is close to human caring since, in everyday practice, it approximates this human experience. As Hanh states,^3^ this is inevitable and unavoidable in people's lives. Buddhism teaches that we should not despair when faced with suffering in our daily lives and the world around us, as is mentioned by this Buddhist quote: "If you want to grow a vegetable garden, you have to bend down and touch the earth."^2^ Likewise, we should "touch our suffering, embrace it and make peace with it".^2^

According to Watson, the challenge of finding new meaning in human suffering is not only the entirety of our task as humans, but it is a professional task in the sense that for a caring-healing practitioner/scientist, the patient and the nursing professional intertwine. Furthermore, the more we search for new understandings that deepen our humanity, the more human, compassionate, wise, and healing we become in our work and world.^1^ In this way, nurses can know the pain and suffering experienced by their patients because they have touched and known the pain and suffering within themselves. Buddhism has said that practicing reflexive compassion creates "constructive karma"^2^ that subsequently affects the well-being of others. Thus, we become a conduit for the human struggle through anatman and empathy until humanity is revealed to all of humanity.^2^

Following what was mentioned previously, Watson recognizes that Caring Science stems from a relational-unitary ontology with the premise that we are all connected and belong to the source, to the universal spiritual field of infinity. This fact clearly shows the connection among all moments in life -such as change, sickness, suffering and the death of loved ones- and in all this, the importance of intentional consciousness, which, along with beauty and peace, become "fundamental elements in human caring" that help to align mind, body, spirit, and integrality. As health and caring-healing professionals, our task is to realize that in our scientific and nursing worlds, our work and jobs have been too narrow for the profoundly human nature of the work we face in our caring-healing relations with Self, others, and our universe.^1^ In her writings, Watson highlights the legacy and history of Florence Nightingale and her practical approach to knowledge -a historical example that still reinforces modern nursing. Her example demonstrates the paradoxical integration of subjective and objective, the connection of accurate data and subjective views, a personal sense of vocation for her mission, and the outer world's work that transcended all the objectivist logic of her era.^1^ This paradigm shift, initially proposed by Nightingale and taken up again by Watson, cites concepts by Palmer, who says that excellent knowledge and great learning should not be done merely in objective terms.^1^^,^^24^ He mentioned that the mythology of objectivism instead involves "power and control over the world or others; that is, it is more of a mythology of power than a real epistemology that reflects how real knowledge operates." In this way, perpetuating this mythology of objectivism does not help us to see that "all epistemology becomes an ethic"^24^ and affects how we value and see the different phenomena in our world.^1^

Despite the pursuit to integrate objective and subjective paradigms, the objective paradigm predominates, as shown by expressions of dehumanized care. Furthermore, while loving-kindness and compassion are considered the cornerstones of nursing practice, certain deficiencies in providing compassionate nursing care can be seen, reflected by the health services offered to the public, not only by service providers but also by policymakers and academics.^25^ The concept of transpersonal relationships, as developed in this chapter, though not original or unique to nursing, stems from the evolution of Watson's thinking and her interaction with the world. Several disciplines and theories feed it, and it has become one of the most essential and central concepts in caring science theory. 

### The transpersonal relationship in caring science theory

In the theoretical proposal of the Caring Science, Watson presents transpersonal caring transactions as those behaviours and scientific, professional, ethical, aesthetic, creative and personalized giving-receiving responses between two people, that is, between the nurse and another, which facilitates contact between the intersubjective worlds that they experience through the physical, mental, or spiritual world.^23^ Watson concludes that it is essential to emphasize the artistic pattern of the transpersonal caring relationship, shown through three aspects. First of all, it is a means of communication and the release of human feelings, which make it possible to progress towards the harmony of spiritual evolution; secondly, it is progressing towards feelings that are more pleasurable to the human being; and thirdly, it is a form of touching the soul, of feeling the emotion and union with the other person.^23^^,^^26^

Three dimensions describe the concept of the Transpersonal Caring Relationship: Self, the phenomenal field and intersubjectivity. Self is transpersonal-mind-body-spirit oneness. It is composed of perceptions with the characteristics of "I" or "my" and perceptions of how "I" or "my" relate to others and various aspects of life. It involves the Self just as it is, the ideal Self that a person would like to be and the spiritual Self. The phenomenal field is the totality of the human experience within the framework of an individual's world, where the subjective reality of the person determines perceptions and responses in given situations, together with objective conditions or external reality. The transpersonal caring field resides within a unitary consciousness and energy field that transcends time, space, and the physical dimension, and it manifests as the unity of the mind-body-spirit-nature universe.^27^ Lastly, Transpersonal Intersubjectivity refers to a relationship in which the person who provides care affects and is affected by the Other. Both are fully present in the moment and feel they are one. They share a phenomenal field that becomes part of their life stories and are co-participants in becoming -now and in the future. This concern for the inner life world and the subjective meaning of the other is fully present.^22^ Those three dimensions are considered integral to human caring. They begin when the nurse enters the vital space or "phenomenal field of the other person" and can connect with the being of the other person (spirit, soul), feeling it and responding to it in a way that provides the opportunity for the other person to express the subjective feelings and thoughts that they have stored away. That is where the intersubjective connection occurs between nurse and patient and becomes the total "caring moment." In other words, it makes it possible to connect with the unique life stories and phenomenal fields that are transferred from person to person.^1^^,^^17^^,^^28^

The key aspects of Human Caring theory are relational caring with a values-based ethical-moral-philosophical foundation; the ten Caritas Processes as the core of caring; Caring as human consciousness-energy-intentionality-presence, Caring-Healing Modalities; and the Caring Field-Transpersonal Caring Moment.^21^ Therefore, exploring the conceptualization of that Caring Moment in more depth is essential. Watson stated that the "caring moment" becomes transpersonal when the nurse and the patient join their life stories and phenomenal fields and become one focal point in space and time, revolving in a more profound and more complex pattern of life.^23^^,^^26^ In addition, the caring moment makes it possible to overcome the nurse's controlling ego, allowing oneself to be guided by compassion in the present and open up to the other through a genuine connection. In that fullness of the present moment, the nurse can "read" the field and go beyond the patient's outer appearance and human responses, thereby seeing and connecting with the spirit of the other.

As a result, this connection between one human being and another enables compassion and caring to expand. It honours the humanity of the other and prevents reducing the other to a passive object.^21^ As Thich Nhat Hanh says, we will always be continually training if we practice this understanding and accept others with their vulnerabilities and suffering. We will learn to transform that suffering into hope, love, and deep compassion (Thich Nhat Hanh 2003), which, as Watson states, is already in our hearts and minds, waiting for us to enter this new place of the consciousness and stay there to transform suffering at the deepest level of life.^1^ The literature on this topic has described behaviours and certain practical aspects-such as humanized, loving, and compassionate caring moments and times- from different professional performance scenarios in clinical, community areas, and teaching and learning. Moreover, patients and caregivers easily identify the qualities that characterize it and aspects of teaching that analyze whether the education professionals receive is adequate for developing these competencies.^28^^,^^29^

Most studies describe compassionate and loving care as the search to connect with patients with needs and suffering experiences. It is also described as giving or having^28^^,^^29^ and conveying clinical information promptly.^15^ Compassionate caring is not a static event; it develops and manifests throughout the hospital stay, becoming more apparent as more visits occur and familiarity with patients grows. The authors of those studies recognize that compassionate health care is a dynamic process that takes place throughout the nursing relationship, highlighting the importance of specific compassionate health care moments while also showing that time limitations do not always offer the opportunity to express compassion outside of situational moments.^28^ Investigations on the concept of Transpersonal Caring Relationships have analyzed caring communication patterns and identified six key elements of communication that support transpersonal caring: being fully present, recognizing the humanity of the human being and treating them as an individual, asking questions and offering clarification frequently, showing flexibility and indicating opportunities while also recognizing challenges.^9^

Various studies have also identified specific relational abilities essential for providing compassionate care, such as getting to know patients, feeling suffering, identifying with them, liking them, and showing respect.^15^^,^^28^^,^^30^ In addition, a compassionate relationship is marked by offering a genuine sense of caring and being willing to provide support. Both patients and health care staff describe a distinct mark of compassionate caring as treating the patient as a person with individual needs. ^15^^,^^28^^,^^31^ This approach involves respecting them for their individuality and their unique situation and respecting and recognizing their beliefs and desires.^32^ Participants in several studies have illustrated that loving-kindness and compassion are shown when doctors and nurses put themselves in the patient's shoes and act out of interest in the other first and foremost.^29^^,^^33^

Some studies also report that compassion is primarily conveyed through attentive, attuned, and conscious listening factors.^2^^,^^29^^-^^30^^,^^34^ Clinical descriptors include feeling or being aware of the patient's suffering ^30^^,^^34^ and non-verbal expressions such as the effective use of silence, listening, posture, tone of voice, visual contact and smiling, which convey a sense of recognition and understanding.^35^ In addition, clarifying or explaining information about the health status of patients and encouraging patients to share their views and feelings about their medical progress have also been mentioned. ^32^ Breneol et al.^36^ analyzed the development of a caring relationship between nurses and children who depended on hemodialysis technology. They found that developing a supportive and trusting relationship can overcome barriers to human caring. In addition, parents who expressed the need for better quality care in the hospital environment identified factors such as the need to be heard and supported, the importance of speaking positive and negative feelings and teachings on transpersonal caring. Some negative aspects identified as inhibitors of compassionate communication are a lack of respect and concern for the other, hostility towards the patient, a judgmental attitude, pity, and incorrect assumptions. In contrast, the patient's caregivers and their families perceive compassion and loving-kindness through qualities such as being present, respect, dignity, kindness and perseverance. In addition, they identify the virtues of caring as honesty, justice, compromise, and the valuable role of compassion at the time of grief.^10^^,^^31^^,^^37^ Other authors have explored cultivating loving-kindness with oneself to mitigate the stress of working with people deprived of their freedom. 

Nurses identify compassion as a skill in their educational process, although they feel ill-prepared to provide compassionate care when transitioning to clinical practice.^38^ Nurses identify compassion as a skill in their education process; however, they feel inadequately equipped to provide compassionate care once they transition into clinical practice.^38^ Considering that experiences in the practice environment significantly affect the nursing student's confidence in incorporating compassion into caring,^10^ it is crucial to remember that compassion can be taught through training by developing and fostering micro-practices that develop and strengthen over time.^10^^,^^28^^,^^30^^,^^39^

### Development of the concept of Transpersonal Caritas Relationships

The present article describes the construct of the Transpersonal Caring Relationship. In this article, we reveal the construct of the Caritas Transpersonal Relationship. Watson developed this concept based on practical experiences implementing caring science in different healthcare settings. It is also the foundation for Watson's Caritas instruments for patients, leaders, colleagues and self-care. When working to validate these instruments, we have found that it is indispensable to have conceptual clarity about the construct and, based on that work, to support nurses, academics and researchers who want to measure the implementation of the theory in different care settings by measuring the Transpersonal Caritas Relationship. Thus, the concept has evolved from a transpersonal caring relationship to a Transpersonal Caritas Relationship, considering that the word Caritas is closer to the practice by nurses who provide care with the theoretical foundation of caring science. As mentioned earlier, Caritas processes^1^ are the core and basis of caring science theory.^1^ These are key nursing processes in the theory's structural core. They provide a universal language for human caring and a basis for the theoretical and philosophical framework. 

"Caritas" means to harbour hope, appreciate, and offer special or loving attention with charity, compassion, and a generous spirit. Caritas processes^1^ provides a set of guiding principles and language for creating and participating in healthcare relationships and settings with patients, along with the technological and clinical knowledge each nurse offers to the person and their professional practice.^1^ To grow in both physical and non-physical dimensions of human caring, a wide range of artistic, aesthetic, spiritual, empirical, political, and ethical forms of knowledge is promoted to achieve a higher level of human connection with the patient.^1^ The assumptions of the Transpersonal Caritas Relationship are a Caritas consciousness, a moral commitment and intentionality on the part of the nurse to protect, improve and potentiate human dignity, integrity, and healing, where a person creates or co-creates one's meaning of existing, living and dying.^1^

The Transpersonal Caritas Relationship is based on the nurse's intentionality and consciousness that affirms the person's subjective spiritual significance while seeking to continue to care amid a threat and despair. This relationship manifests through the harmony and unity of mind, body and spirit, through a relationship of support, love, compassion and trust between the receiver and giver of care. Intentional presence is defined as a conscious and altruistic choice born from moral virtue and disinterest. It aims to act reflexively, empathetically and in a humanistic manner that honours and gives meaning to each person's uniqueness and the caring-healing-nurse-patient interaction. The nurse who responds to the patient's needs with loving-kindness to alleviate a real or perceived threat to personal integrity alleviates suffering. This response requires integrating scientific and humanistic paradigms, both essential to caring.^2^

It is also important to mention that the Transpersonal Caritas Relationship honours the I-You relationship instead of an I-That relationship. It is a communication method that stems from a mutual understanding and desire between the caregiver and the care receiver. The nurse seeks to recognize, detect, and connect with the inner condition of the other's spirit through a compassionate and genuine presence centred on the caring moment. In response to the patient's suffering, they connect to inherent qualities through recognition, commitment and action. The Transpersonal Caritas Relationship is determined mainly by its fundamental attributes, nurturing or eroding in clinical and educational settings.^10^ It is also worth highlighting that the connection established during the Transpersonal Caritas Relationship is achieved through actions, words, behaviours, cognition, body language, feelings, intuition, thoughts, senses, and the energy field. The nurse shows the ability to connect with another person at this spiritual, transpersonal level through movements, gestures, facial expressions, touch, sound, verbal expressions, procedures, information, and other means of communication, including scientific, technical, aesthetic and human, which transform into art or acts of human caring or modalities of intentional caring-healing.^26^

Caring-healing modalities in the Transpersonal Caritas Consciousness improve the harmony, integrity and unity of the being and release blocked energy that interferes with natural healing processes. The nurse guides the other towards accessing their inner healer by helping them relate correctly with their spiritual source through self-care, self-knowledge, self-control and self-healing.^17^ Under this construct, the nurse's professional development and their spiritual practice enable them to enter a deeper level of professional healing practice, which facilitates their evolution and their awakening to the transpersonal condition of the world, as well as their becoming a Nurse Caritas who cares, who maintains a Transpersonal Caritas relationship with themselves and others.

In contrast to what has been mentioned, it is crucial to recognize that nursing faces multiple obstacles to achieving a transpersonal caring relationship while giving care, such as exhaustion, external distractions, difficult patients or families and complex clinical situations.^40^ As a result, it is vital to keep in mind that if loving-kindness and compassion are considered to be essential to developing professional identity, and working with powerful emotions and experiences broadens personal consciousness, then developing an environment that contributes to empowering and fostering resilience is a priority for developing and maintaining compassion. Furthermore, models that can be followed and a corporative team spirit are needed to train nurses in compassionate behaviour.^41^ This need for training is the main reason why opportunities for self-reflection should be fostered in the field of education, with empathetic professors who can evaluate students and help them take responsibility for compassionate caring and who can create teaching environments that emphasize competencies based on the approach of working with the emotions and related knowledge.^42^

Some educational interventions for incorporating loving-kindness and compassion in the clinical setting involve the health professional and the patient producing artistic expressions such as music, theatre, literature, and expressive writing. In addition, clinical simulations improve training, engaging in reflexive practices for improving self-awareness, training in communication skills, participating in institutional activities that increase work satisfaction and enhancing competencies in providing care.^42^ In clinical areas where the critical patient is treated, behaviour by health staff can help patients and their families feel supported through positive behaviours, which can contribute to effective mental health and recovery outcomes and trust in the health care team.^43^^-^^44^ Since age can be a factor in exercising compassionate caring, it is essential to design strategies for the healthcare staff to develop this aspect. For example, the study by Basile found that younger nurses were perceived as more compassionate than older nurses.^43^ Lastly, as mentioned earlier, this Transpersonal Caritas Relationship construct comes alive in nurses' daily practice, theoretically grounded in caring science. It seeks to make its premises evident within the reality of the environments in which nurses establish relationships with themselves, the patient, their colleagues, the environment and the universe.

## Discussion

Watson has shown the philosophical evolution of her concepts over time. The article herein proposes a conceptual paradigm that describes and reconsiders the Transpersonal Caring Relationship construct in terms of the concept of Caritas, which means to harbour hope, appreciate and offer unique or loving care with charity, compassion, and generosity of spirit. The investigation becomes a challenge, due to which the effectiveness of different strategies for improving the evolutional level of the Transpersonal Caritas Relationship in caring for people continue to be evaluated in various settings, including educational, clinical and community. 

During these times, when human nature is facing a pandemic that has brought about abrupt changes in lifestyles and brought us closer to illness, death, and collective suffering, it is imperative to employ a philosophical nursing framework based on the transpersonal relationship in which loving-kindness and compassion are the focus of healthcare. In addition, the caring science approach could mark a turning point amid the progressive loss of sensitivity to humanism that has resulted from the predominance of the scientific-technical paradigm, levels of evidence, order, prediction, control, methods, generalization, separation and objectivity.^11^ This debate between scientific-technical versus transpersonal is accompanied by numerous barriers that have been identified related to a health system that reduces the potential for loving-kindness and compassion when caring for patients due to the lack of time, support, staff, and resources.^13^^-^^15^^,^^45^ Similarly, a "production line" mentality exists^13^ in which the economic approach considers administrative functions such as paperwork, litigation, metrics, and efficiency to be primordial, which distances clinicians from the patient's bedside where the transpersonal relationship can be more easily experienced. Additional barriers include a negative organizational and work culture with resistance to change and entrenched opinions and attitudes on the part of the staff. ^30^^,^^38^^-^^39^^,^^45^

With the approaches presented herein, this article introduces a transitional adaptation from the concept of the transpersonal relationship to the transpersonal Caritas relationship, which is the basis for Evolved Caritas Nursing. This evolved concept recognizes that the mutuality of caring affects the universal field to which we all belong and that the Caritas consciousness and actions affect the energy field that is present when the nurse relates to the other, enabling the compassionate heart to awaken, permitting nurses of the new era to access their spiritual nature and that of each person, and to connect with the source of universal love. In this way, they evolve into the Caritas consciousness, making it possible to recognize the other with loving-kindness in the practice of caring.

To conclude, the poem cited by Watson in her “Unitary Caring Science: The Philosophy and Praxis of Nursing,” published in 2018,^1^ invites us to reflect on the pursuit of inner growth: "While from the bounded level of our mind. Short views we take, nor see the lengths behind, but more advanced, behold with strange surprise. New distant scenes of endless science rise! ".. Alexander Pope.
